# Decision Maker Profiling Using Their Mental Behavior Pattern

**DOI:** 10.3389/fpsyg.2021.667255

**Published:** 2021-08-18

**Authors:** Flávio Luis de Mello, Sebastião Alves de Souza

**Affiliations:** ^1^Electronic and Computer Engineering Department, Polytechnic School, Centro de Tecnologia, Ilha do Fundão, Federal University of Rio de Janeiro, Rio de Janeiro, Brazil; ^2^Familiacomvida Clinic, São Paulo, Brazil

**Keywords:** degree of differentiation of self, systemic-linking method, cognitive interactive pattern, artificial intelligence, decision making

## Abstract

This study describes a method to assist the task of predicting the result of the decision-making process of an individual based on psychological and emotional aspects and using artificial intelligence (AI) techniques. This study presents indicators created for profile identification, which are organized in primary and circumstantial categories. These indicators are merged according to the ultimate purpose of profile identification, including the expected behavioral pattern for a person who performs a decision-making process. The person behavior hypothesis was successfully tested and can be approximated by an indicator such as mental functioning pattern, and the mental functioning pattern hypothesis can signal the most likely decisions of an individual. Four debtor decision variables were assessed in a debt negotiation process, in order to validate the method, which is applicable to other decision-making domains. The best signaling of the most likely decision of the debtor was seven times greater than that of a random prediction, while the gain of the worst decision signaling variable was 20%.

## Introduction

This study presents a procedure to identify the profile of a decision maker based on psychological and emotional aspects and using artificial intelligence (AI) techniques. The procedure includes indicators created for profile identification, which are organized into primary and circumstantial categories. Such indicators can be merged according to the ultimate purpose of profile identification, including the expected behavioral pattern for a person who performs a decision-making process. The psychological analysis follows the concept of the mental functioning pattern (Mello and Souza, 2019), while the technique of classification into profiles is adjusted by unsupervised machine learning.

The search for a relationship between basic emotions and the behaviors of individuals in the face of difficulties is a fascinating line of a research study. A possible approach is to observe the phases of childhood, adolescence, pre-adult, adult, and elderly using an inductive method while always trying to correlate the emotions learned and internalized by that person with his/her behavioral manifestations today. This procedure is used here to find what is called the specific mental functioning pattern of an individual. This specific pattern is defined as the composition of the historical ties of the family of the person, and his/her previous generations, with the expression of his/her uniqueness and socialization through his/her behavioral and emotional repertoire. The search for this specific pattern is examined according to cognitive, affective, and behavioral domains, the purpose of which is to find points that can serve as indicators in the decision-making process of an individual and in the act of making choices when there is a need to solve problems. Identifying a specific pattern of a person increases the ability to predict his/her actions, which facilitates the task of inducing his/her behavior, and enables the nudge resource to be put into practice (Thaler and Sunstein, 2008), which helps in patient awareness work. Therefore, knowing the specific mental functioning pattern is a useful action for the clinical area and for all other areas of psychology.

The decision-making process is far from being merely rational, having a significant emotional factor. At the moment of decision-making, rationality shares importance with several automatic processes of human behavior and with the perceptions, judgments, and emotions of an individual. In addition, the human mind tends to be lazy when it comes to understanding sophisticated information. Simon (1979) studied how a person organizes a complex mass of information to formulate a problem that reduces its efforts in the search for a solution, starting from a certain stimulus environment and a given wealth of knowledge. Such a mind often seeks to minimize the effort of thinking, triggering processes already known to it, usually automatic, homeostatic, and healthy or not. In this way, understanding the way individuals have learned to interpret things in the environment in which they live is fundamental to understanding their decision-making.

The main contribution of this work is to describe a unique method that assists the task of predicting the outcome of the decision-making process of an individual. Thus, on the one hand, the objective here is to describe a method of prediction that identifies a personality profile and a model of functioning of a person. On the other hand, it is not part of the scope of the work to classify behaviors as normal or pathological. The profiling task is performed by assessing cognitive, affective, and behavioral characteristics that are expressed in the degree of differentiation of Bowen (1991). In this sense, the next section presents works related to the object of study and that somehow contributed to the design of this method. The subsequent section describes the method of obtaining the specific mental functioning pattern, calculated from cognitive, emotional, and behavioral characteristics, which are grouped according to primary and circumstantial categories. In a further section, the technique of pattern classification using machine learning is presented. Next, a section is presented describing an experiment to verify and validate the accuracy of the proposed method. The last section deals with final conclusions and relevant notes arising from this study.

## Related Works

Simon (1959) made important contributions to the study of the influence of emotional factors in the decision-making process in an economic environment. This author noted that knowledge of perception, cognition, and the environment in which a decision is made is fundamental in order to understand human decisions. The author also emphasized that studying only the goals of the decision maker provides a limited analysis and that if the environment presents changes, if there are multiple objectives, or even if the organism is afflicted by internal conflicts, this approach is not promising. In other words, Simon (1979) maintained the existence of more elements involved in decisions than the maximization of the utility function can explain. The author also noted that if alternatives are not initially provided to the decision maker, then the decision maker has to study them (Simon, 1979). The decision maker creates a mental image of how good an alternative must be, and as soon as a possibility is discovered that is in line with this level of aspiration, the decision maker would finish the study and choose the alternative. This process became to be known as satisficing and ultimately uses personal experiences to build an expectation of how good the solution to a problem should be. According to this approach, whether due to limitations of cognitive capacity, time constraints, or effort saving, agents use rules of conduct (heuristics) to achieve a certain level of satisfaction (not optimization). There is a reasonable consensus in contemporary psychology that human beings, when faced with complicated or ill-defined situations, use characteristic and predictable methods of reasoning. Reinforcing the work of Simon (Ranyard, 2017; Nørgaard, 2018; Gangl and Kirchler, 2019), psychology has shown that, on the one hand, the human being uses deduction only moderately and, on the other hand, is very good at seeing, recognizing, and combining patterns. Thus, in the face of complicated problems, an individual observes patterns and simplifies those problems. The feedback received from the environment has a role in strengthening or weakening these constructions and, thus, reinforces their use or leads to their disposal, something like rules of thumb.

Following in the footsteps of Simon, Hands (2001) suggested the AI approach to search selectively, employ rules of conduct, and stop the search when a satisfactory solution is found. Basov et al. (2007) proposed caution heuristics according to which some individuals with a more naive position always choose the safest option in the face of more risky or uncertain situations, and those with more sophisticated positions choose to compute the expected utility of each state when facing binary choices. Bobadilla-Suarez and Love (2018) also claimed that heuristics are simple, yet effective, strategies that people use to make decisions. Shrestha et al. (2019) reported the idiosyncrasies of human and AI-based decision-making along with five key contingency factors: specificity of the decision search space, interpretability of the decision-making process and outcome, size of the alternative set, decision-making speed, and replicability. But it is Camerer (2017) who made two important statements: Human prediction is imperfect machine learning and human judgment is like overfitted machine learning. These studies suggest that a systemic and heuristic approach can be used to assess a behavioral profile when making a decision.

Many studies (Hawkins et al., 2008; Durlak et al., 2011; Weissberg et al., 2015; Domitrovich et al., 2017) have indicated that people with high levels of socio-emotional skills have greater self-esteem, self-efficacy, greater persistence toward goals, better interpersonal relationships, and greater commitment and school performance. In the long run, higher levels of skills are related to higher educational levels, greater professional success, more positive family and work relationships, better mental health rates, reduced psychopathology, and lower levels of conduct problems. These skills become competencies when the individuals reflect on the decisions that they will make in the face of situations in which a choice is to be made. It is understood here in this study that if these skills and competencies can be properly chosen as metrics, then there is a set of indicators capable of signaling the profile of an individual, something that is explored in depth herein. By evaluating these indicators separately, one can more accurately capture the way an individual learned to interpret things in the environment in which they live, thus allowing the method presented in this study to remain aligned with the mental images originated by neural patterns of (Damasio, 1994; Kirkebøen, 2019) and with prospect theory (Kahneman and Tversky, 1979; Tversky and Kahneman, 1992; Zhang et al., 2018), which considers the psychological factor in decision-making, which involves the various processes of human behavior and their perceptions, judgments, and emotions.

Bowen (1978, 1991) noted that people differ from each other according to their mental functioning and developed his observations under the concept he called the differentiation of self-scale. The scale is a way of evaluating all people along a single continuous line in time, ranging from the lowest (0) to the highest (100) possible degree of human functioning, which can be compared to a scale of emotional maturity. This scale is fundamental in this study because it eliminates the concept of normal, which, in terms of psychiatry, has no precise definition and has nothing to do with the concept of emotional illness or psychopathology. Based on this scale, it is possible to map and understand the mental functioning pattern of a person at the time of decision-making when manifesting emotions consciously or unconsciously.

Lampis et al. (2019) compared a normative sample and a sample of adults seeking services at a systemic therapy clinic and suggested that information about differentiation of self might enable counseling psychologists and therapists to define effective interventions. Messina et al. (2018) examined the impact of counseling on Bowenian differentiation of self. Kudo (2018a,b) reported on a study of feasibility and reliability of profiling, anticipated by the differentiation of self- scale, and confirmed by anamnesis performed directly with the patients. Vargas et al. (2018) correlated the instrument for measuring differentiation of self with Gordon's personality instrument.

## The Profiling Method: Specific Mental Functioning Pattern

According to Watzlawick et al. (2014), the search for a pattern, or a model, is the basis of all scientific investigation. Where there is a pattern there is a meaning, this epistemological maxim is also valid for the study of human interaction. According to Bowen (1991), behavioral and interactional sequences of an individual, or a group, have characteristics of repetition, reflect the emotional system they possess, and are reactions to certain environmental conditions. This suggests that all behavior is the result of interaction and changes in the scenario where the individual is inserted. Thus, a relationship that is repeatedly established between conditions and behaviors gives rise to a certain degree of predictability.

The construction of the basic links necessary for the survival of individuals, by means of their interactional dynamics and adaptation processes, forms affective behavioral patterns. A change in the behavior of an individual, or of a member of the group, affects others at the same time and is affected by similar changes. This format of interaction is called the mental functioning pattern (Mello and Souza, 2019), which seeks to investigate, map, and understand the meaning attributed by a person when experiencing various episodes, facts, or events during the various phases of his/her life cycle. These events contribute to the construction of historical baggage of individuals used in interactions with family, friends, and in other relational contexts in which they are inserted.

The mental functioning pattern is modus operandi as organisms learn, think, and decide, being built from organic and cognitive structures. Organic structures are anatomical constructions originating from hereditary and biological pre-dispositions plus the learning acquired by an individual during interactions and exchanges of experiences with family and the social environment in which an individual is inserted. According to Kandel (2006), the functional anatomy of the cerebral mechanism of a living organism, in relation to its perceptions, emotions, and behaviors, is basically based on the organic structures formed from synaptic and neuronal connections. These synaptic connections form short-term memories when activated by effective and practical stimuli; that is, they allow a type of temporary learning, which Kandel calls “functional changes.” The significant stimuli of synaptic connections, when repeatedly received by a neuron for several weeks, potentiate the efficiency of these synapses, which over time start to form a new circuit of neurons, form long-term memory that Kandel calls “structural changes.”

Cognitive structures, on the other hand, are representations of the way living organisms perceive, get emotional, and behave (Maturana, 1975). The cognitive structures of a person are formed by hereditary and biological predispositions complemented by learning acquired during his/her interaction and exchange of experiences with family, the social environment, and cultural circumstance, and new information he/she receives from the environment in which he/she is inserted. Bateson and Ruesch (2006) argues that if culture and new information are not powerful enough to deconstruct the mental functioning pattern of an organism, whether through addition, repetition, or change in perception, the living organism will probably not adapt or evolve.

Note that this study establishes a causal relationship between the mental functioning pattern of an individual and the decisions he/she makes. In other words, this study uses the hypothesis that a decision is associated with the mental functioning pattern. Note that such causality is not a function of truth and is at most a probability that a particular way of deciding will be accompanied by a type of mental functioning pattern. Thus, this pattern is an explanatory hypothesis that best explains the decision of an individual. The construction of a plausible hypothesis about an unexplained fact is the definition of an investigative method known as abduction (Peirce, 1997–CP 2.98). According to Pierce, the stages of any and all scientific research studies necessarily take place in the following order: abduction, or discovery of a hypothesis; deduction, or extraction of the consequences of the hypothesis; and induction, or hypothesis testing. It is a process of acquiring knowledge, whether it is automatic or not (Mello and Carvalho, 2015; Oliveira et al., 2020). For this reason, this study presents arguments that justify proposing the mental functioning pattern as a hypothesis to explain decisions made by an individual. It then shows deductive inferences that corroborate the hypothesis, in order to finally subject it to testing.

In this study, the search for the identification of this pattern occurs through questions in an attempt to investigate or, at least, approach the pattern that links the persons to their family relational context and environment. The concept of context, according to Bateson and Ruesch (2006), is linked to another indefinite notion called meaning: “.without context, words, and actions have no meaning.” Having an explanatory hypothesis associated with the pattern that links living organisms to the ecosystem, we seek to obtain a classification of the mental functioning pattern of a person based on the cognition process (in the sense of perceptions, emotions, and behaviors), that is, the process of living of a person (Maturana, 1975).

This attempt to find the mental functioning pattern, through questions about various relational experiences that were possible to be lived, makes it possible to create hypotheses about a person degree of differentiation, autonomy, assertiveness, and self-esteem in relation to affective/relational, cognitive, and behavioral qualities. With such information, a person is assigned a predicate corresponding to the way in which organisms or aggregates of particular organisms learn, think, and decide. Thus, as a way to classify, systematize, and find the mental functioning pattern of an individual, three types of degree of differentiation of the person were studied (Bowen, 1991): undifferentiated, moderately differentiated/undifferentiated, and differentiated. These competences can be measured or evaluated through his/her interactions within the family and in the environment, using a model similar to that used by Bowen (1978). This method uses a differentiation scale that attributes some personality characteristics to the individual and measures the degree of differentiation on a scale from 0 to 100.

An undifferentiated person tends to have great difficulty with putting into practice his/her affective, cognitive, and behavioral skills and potentialities, which forces him/her to subordinate himself/herself and adapt to the environment in which he/she finds himself/herself. A moderately undifferentiated, or a moderately differentiated, person tends to exhibit a position of indecision between situations that bring expected results and situations whose results are uncertain or have never been experienced. Finally, a differentiated person tends to be realistic, creative, and decisive when putting into practice his/her skills and potential, even knowing his/her rational and emotional limitations. This study proposes to characterize the differentiation of an individual simultaneously according to indicators of adaptation, reactivity, and creativity, each characterized by behaviors associated with a way of making a decision (the individual receives a trio of values associated with their behavior). Note that human functioning is dynamic, so a person may have predominantly adaptive characteristics, for example, and occasionally have reactive and creative attitudes, which justify the use of a trio of values, instead of a single one as proposed by Bowen.

The concepts of adaptation, reaction, and creativity are described by Mello and Souza (2019). According to the pattern of adaptation, the mind tends to accommodate in order to continue in comfort, to preserve pleasure, and to avoid pain or suffering, something that is manifested through mechanisms of anxiety and depression. The mind molds itself to the circumstances to avoid effort and energy consumption. The second pattern is that of reaction, which concerns a mind that struggles to return itself to well-being, while in the conflict between leaving the old comfort zone to find a new one, the symptoms of which are aggression or phobias. Finally, the pattern of creativity concerns the ability of an individual to learn and make solutions possible by creating new possibilities and alternative meanings for situations of displeasure, pain, suffering, and discomfort. The perception of a patient is amplified when facing difficulties, and thus, new information is more easily assimilated.

The proposal described in this study uses a scale from 1 to 10. Unlike the more relaxed Bowen criterion, which uses a single metric to score the personality of an individual, the proposed method uses two sets of metrics associated with affective, cognitive, and behavioral manifestations, which express the way that individuals learn, think, and decide (Bateson and Ruesch, 2006). In this sense, it is important to understand that this study uses the classic concept of metric and indicator (McClave and Sincich, 2005). A gross measure is understood as a metric, that is, atomic data (e.g., grade 9 for the anxiety metric). The indicator is that which signals a specific situation, that is, it aggregates information according to a context (e.g., anxiety > 8 indicates a propensity to panic and phobias).

In general, a psychologist conducts a semi-structured interview to produce a survey of the personality characteristics of a person. Several behavioral and emotional patterns learned throughout the life cycle can be roughly measured during this process of anamnesis. In this study, each evaluated characteristic belongs to a set of metrics called a category: primary or circumstantial. Therefore, a category is a set of cognitive, emotional, and behavioral characteristics inherent to a group of people, which are manifested in their subjectivity. Kant (2008) characterizes the elements that make up a category as concepts, which allow the multiplicity of disordered sensations of an experience to be reduced to an intelligible unit, thus allowing knowledge. Therefore, the categories define general concepts that express relationships, aspects of interest, and human behavior.

The primary category, on the one hand, is associated with universal, innate, and pre-organized characteristics (Damasio, 1994). In this way, it constitutes a set that contains the same metrics, whatever the objective of the analysis, since such characteristics are always present. The circumstantial category, on the other hand, groups social characteristics, which are activated by more complex emotional behaviors developed at the beginning during the birth of human beings and shaped according to their experiences throughout existence. These social characteristics may be relevant in certain analyses and unnecessary in others, according to the context to be studied. For example, in a credit analysis circumstance, there is a specific interest that is not shared in a profession advising circumstance. Thus, considering the objective of the analysis, a subset of metrics appropriate for the analysis to be carried out is extracted from the circumstantial category. Table 1 lists the metrics covered in this study, the category to which each belongs, and a breakdown of their meaning.

**Table 1:** Description of metrics associated with affective, cognitive, and behavioral manifestations.

**Characteristic (metric)**	**Category**	**Description**
Anxiety	Primary	A state, or organic condition, independent of any specific stimulus that manifests itself in the form of sensitivity, reactions, and behavioral responses
Self-discipline	Primary	The ability of a person to put into practice the actions that make their goals and objectives possible
Self-esteem	Primary	Awareness of the qualities, attributes, and competences that a person believes to have value, manifested through self-significant attitudes, beliefs and emotions, and behavioral manifestations
Beliefs	Primary	The way the subject knows, perceives, and acts from his/his values and purposes, interfering in the affective, cognitive, and behavioral domains
Cultural empowerment	Primary	Discernment about the strength and power that culture exerts when making decisions
Cognitive flexibility	Primary	The ability to change beliefs, paradigms, and values in the face of a process of transformation and adaptation. It is about autonomy to demystify common sense. It is a functional process that acts within the scope of synaptic connections in order to signal the need for change
Cognitive preservation	Primary	The ability to use perceptions, emotions, and behaviors of an individual to build knowledge that helps him/her adapt to the environment and evolve
Learned resilience	Primary	The ability of a person to acquire from experiences lived, transforming them into learning, so that these can help the person overcome adversities and restore his/her personal and inter-relational balance with a focus on his/her life project
Financial literacy	Circumstantial	The learning acquired by a person regarding how to work, balance, and economically plan his/her expenses and income
Family environment	Circumstantial	The learning acquired from living with family culture, which makes it possible to make decisions and choices
Risk attachment	Circumstantial	Refers to people who consciously make decisions that challenge empirical and scientific evidence beyond the limits of what would be a risky behavior, despite the fact that this evidence recommends analyzing, preventing, and avoiding such decisions. Such people act despite being aware of the high probability of putting their lives, and those of others, at risk while deciding to take risks
Assertiveness	Circumstantial	The emotional competence that determines that an individual can take a clear position, that is, he/she is not undecided on what to adopt
Self-control	Circumstantial	The behavioral manifestation of an individual in which he/she use the balance between reason and emotion to define the best scenario for him/her to act
Self-image	Circumstantial	The coherent relationship between a person's image of himself/herself and that which others have of them. It has a descriptive character of how the person is or imagines him/her to be
Rational numbness	Circumstantial	The abusive use of logical reasoning to infer, deduce, solve problems, make decisions, or make choices in the face of facts inherent to the human condition, which cannot be scientifically proven by means of the basic principles that underlie a rational decision
Affective flexibility	Circumstantial	The ability of a person to change perception, emotion, and behavior to solve problems that arise during his/her personal, interpersonal, marital, family, and professional life
Degree of education	Circumstantial	The quality of information of individuals and how they structure their formal reasoning
Degree of experience	Circumstantial	The ability to have an improved view of reality, not dealing with intellectuality of an individual. It is the repertoire of experiences that allows a better adaptation to the circumstances around an individual
Emotional imbroglio	Circumstantial	The attempt of a person to try to compensate for his/her past material and emotional losses at the present moment of decision-making
Emotional immunity	Circumstantial	The ability of a person to learn from his/her own experiences and the experiences of others, transforming them into resources, tools, and skills to preserve himself/herself in the circumstances that life imposes on them
Impulsiveness	Circumstantial	The reactive process of an individual in the face of situations, exhibiting behavior characterized by little, if any, forethought, reflection, or consideration of the consequences
Chaotic insensitivity	Circumstantial	The difficulty for a person to perceive the relational context in which he/she is inserted, which makes it impossible to interpret, predict, and decode the real, imaginary, and symbolic experiences that are exposed through descriptive, narrative, and dissertative texts or through another language code relevant to that exhibition
Family loyalty	Circumstantial	Measures how expectations of a family are managed to achieve the goals one wants for himself/herself. The idea here is to detect whether the person is going to fulfill a family mission that was given to him/her, which in general can hinder his/her own decision
Cerebral plasticity	Circumstantial	The ability of a person, in the face of change, to assimilate new information necessary to his/her condition of survival
Emotional task-fulfilling	Circumstantial	The ability of a person to go through various traumatic experiences, or not, without being able to internalize them, and then to repeat them insistently in new similar episodes
Affective transitivity	Circumstantial	The ability of an individual to relate to different groups of people and to move between relational contexts, without losing his/her identity matrix built from his/her individualization and his/her feeling of belonging

Note that if the metrics are associated with affective, cognitive, and behavioral manifestations, then they model phenomena that have indeterminate limits. For example, it is not trivial to define the borderline value that separates low anxiety from high anxiety. Likewise, the definition of such limits for any other behavioral characteristic (metric) addressed in this study is also unclear. Fuzzy logic is a line of research study within mathematics that aims to deal with this type of situation where there is a classification problem that demands dealing with inaccurate variables (fuzzy variables). Thus, features are valued qualitatively using linguistic terms and quantitatively using a membership function.

In this study, we propose to use the native way of modeling fuzzy logic characteristics in the construction of metrics associated with the manifestations of an individual. Thus, there are two distinct moments during the acquisition of data about the individual: (1) Linguistic terms (qualitative modeling) are used to present questions to a person, who qualitatively chooses his/her answers within a scale of 1 to 10; (2) a pertinence function (quantitative modeling) is used to map the chosen response value on the degree of the pertinence of perception of this person with the indicators of adaptive, reactive, and creative behavior. Table 2 presents an illustration involving the anxiety metric.

**Table 2:** Qualitative and quantitative scheme used to collect and model the responses of an individual.

Question: Are you a person that is in a constant state of alert?	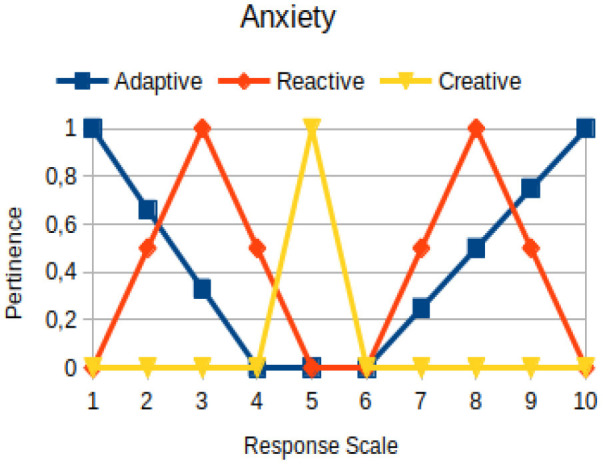
	
	
	
Response scale:	
	
No 1 2 3 4 5 6 7 8 9 10 Yes	
	
At times	
	
	
	
	
	
	

The construction of the pertinence functions for each behavioral assessment metric (Table 3) was obtained from a field research study in a psychotherapy clinic and interviews with psychotherapists familiar with the mental functioning pattern method (Mello and Souza, 2019). For each pattern (adaptive, reactive, and creative), psychotherapists were asked what the response would be of a patient in line with the pattern in question, what would be the response for those patients with no alignment with the pattern, and whether that peak of alignment would be maintained for some response values or does it decay immediately after reaching it. There were many pertinent functions with the reactive pattern showing a saturation at its apex, which translates into its trapezoidal shape when graphed. This is because, in this case, a person tends to take a long time to promote emotional changes, which translates into certain stagnation.

**Table 3:** Pertinence functions for the evaluated metrics.

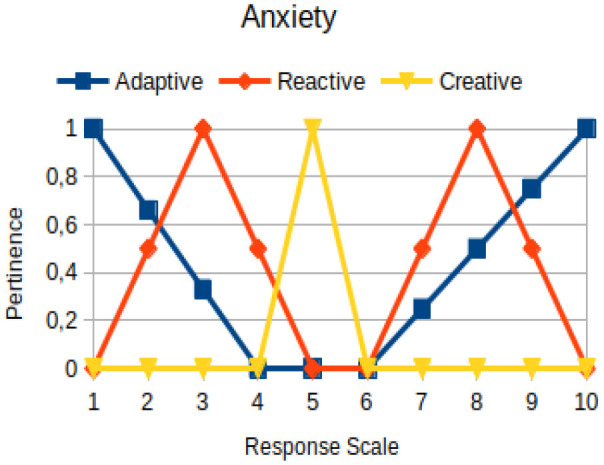	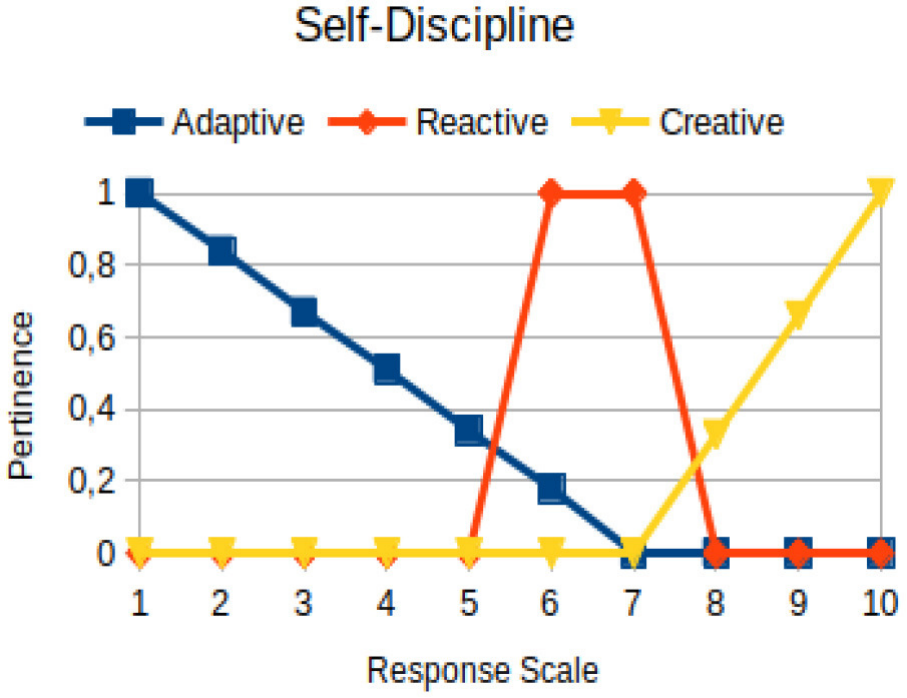
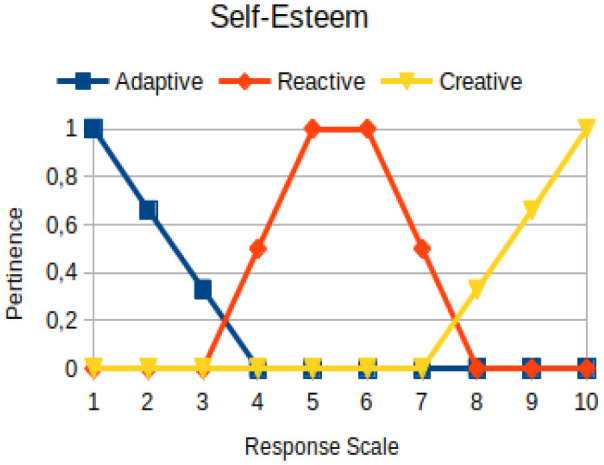	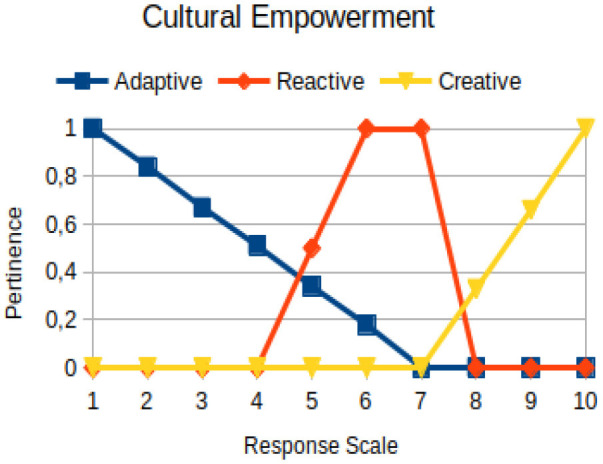
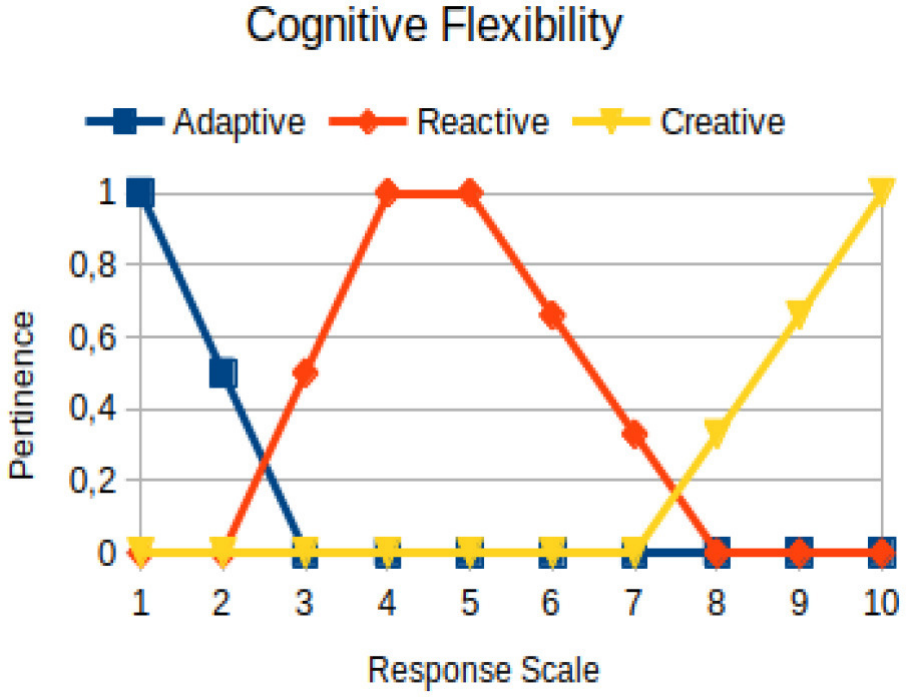	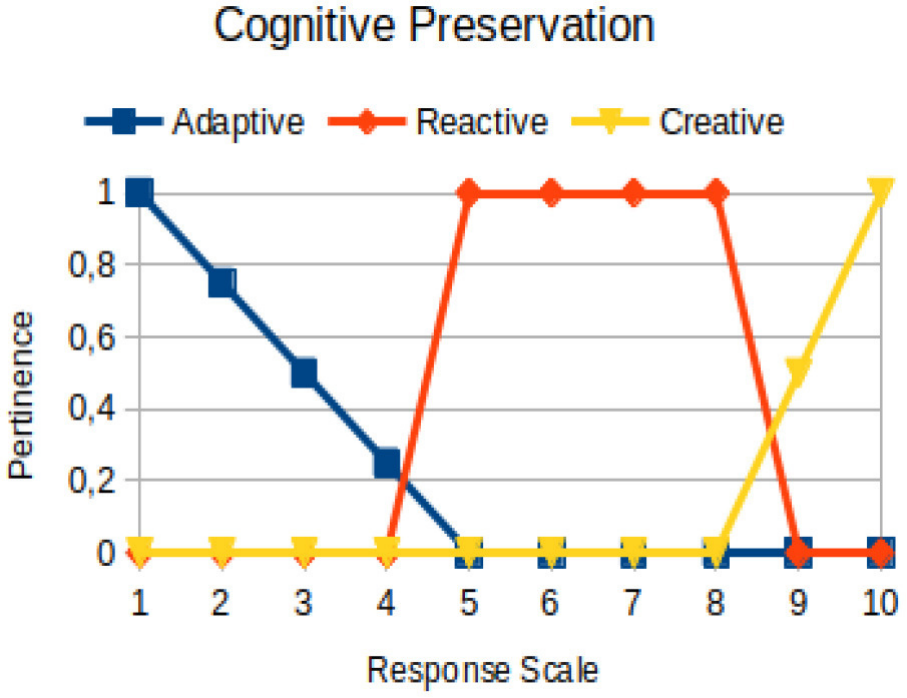
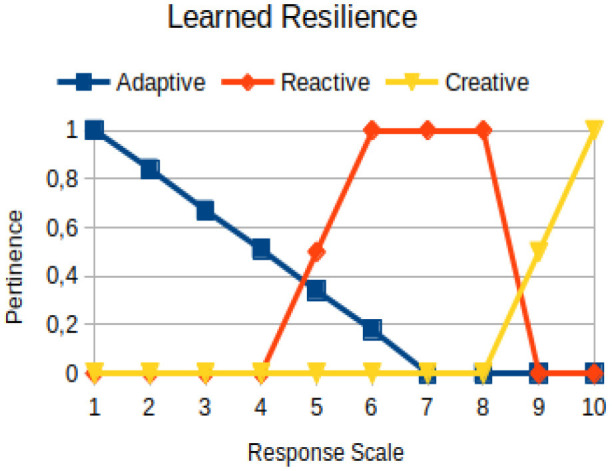	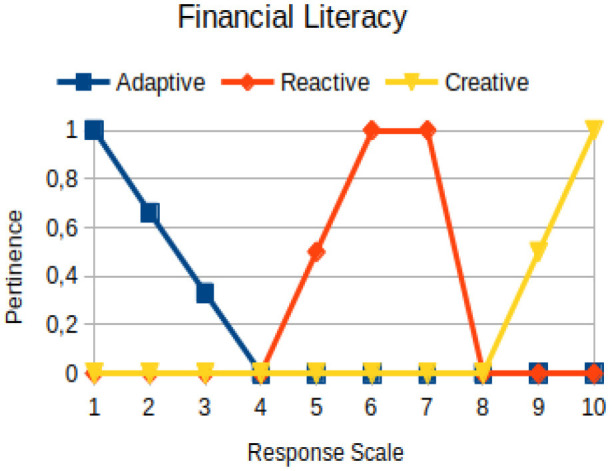
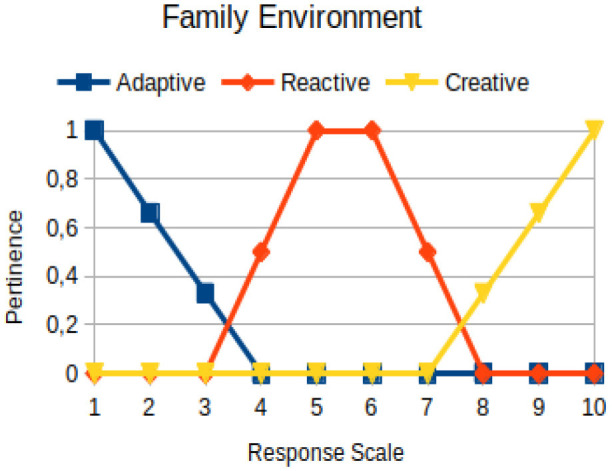	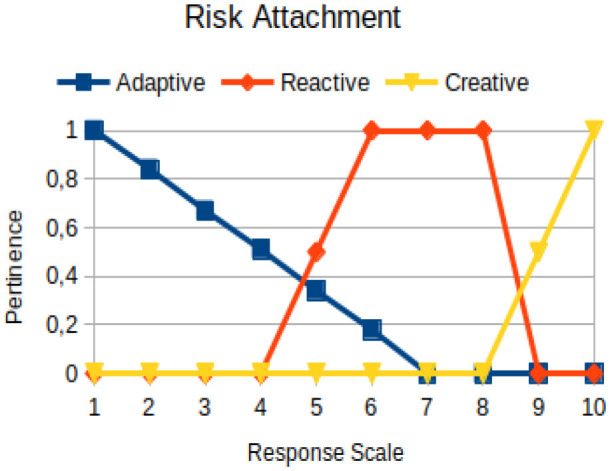
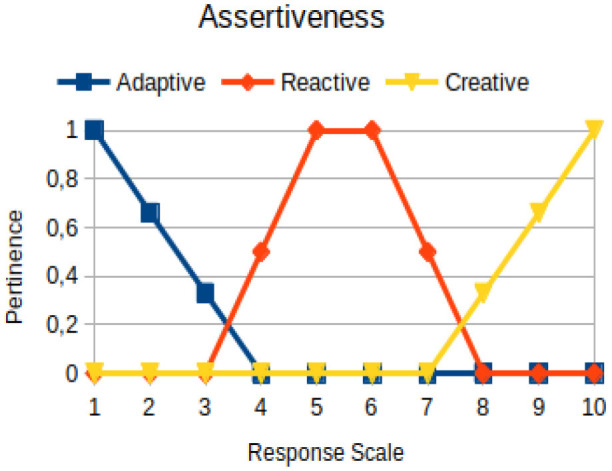	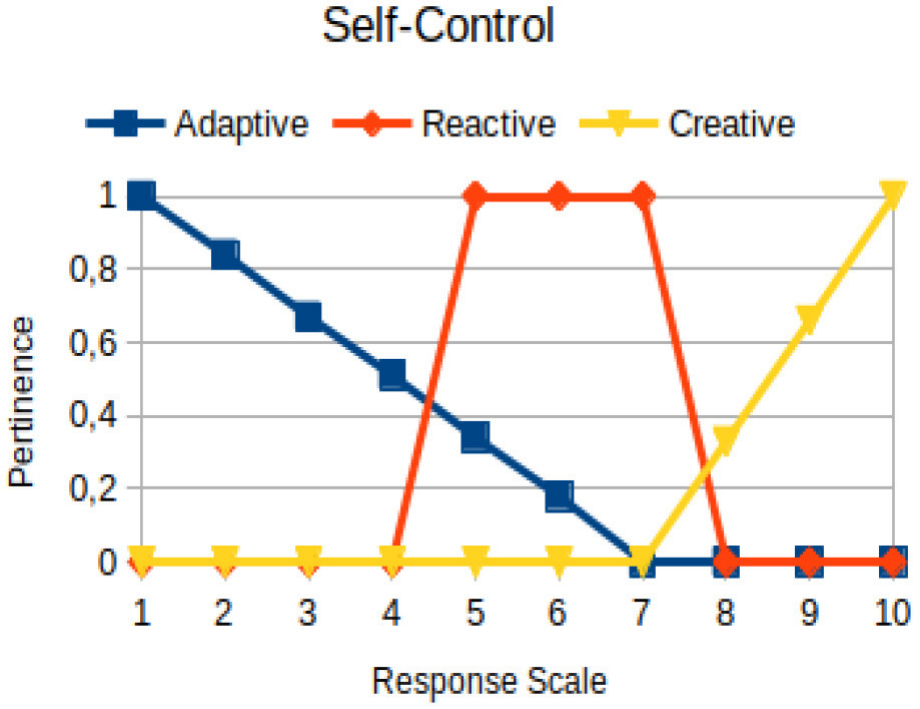
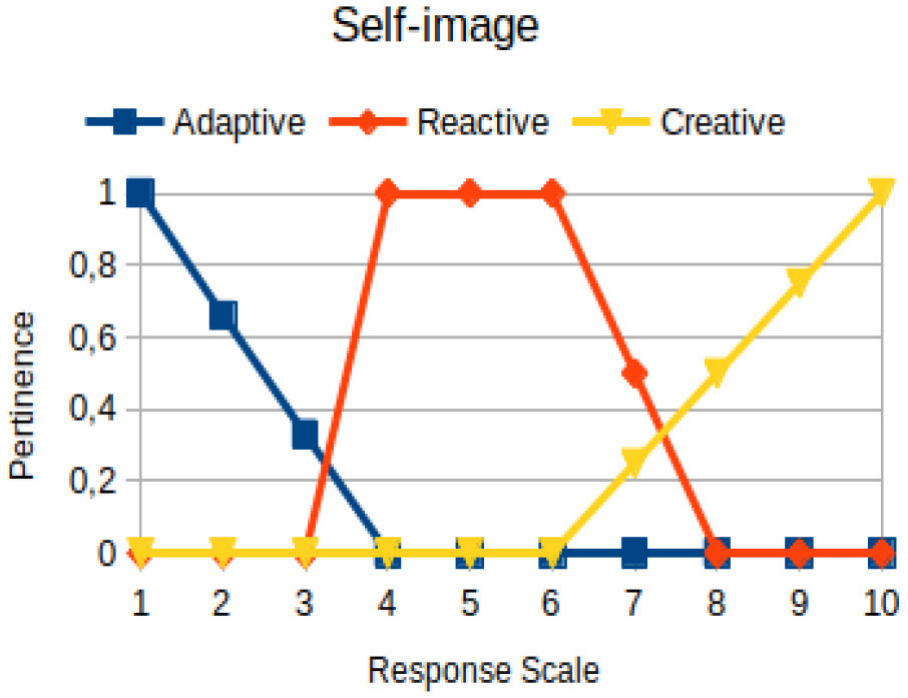	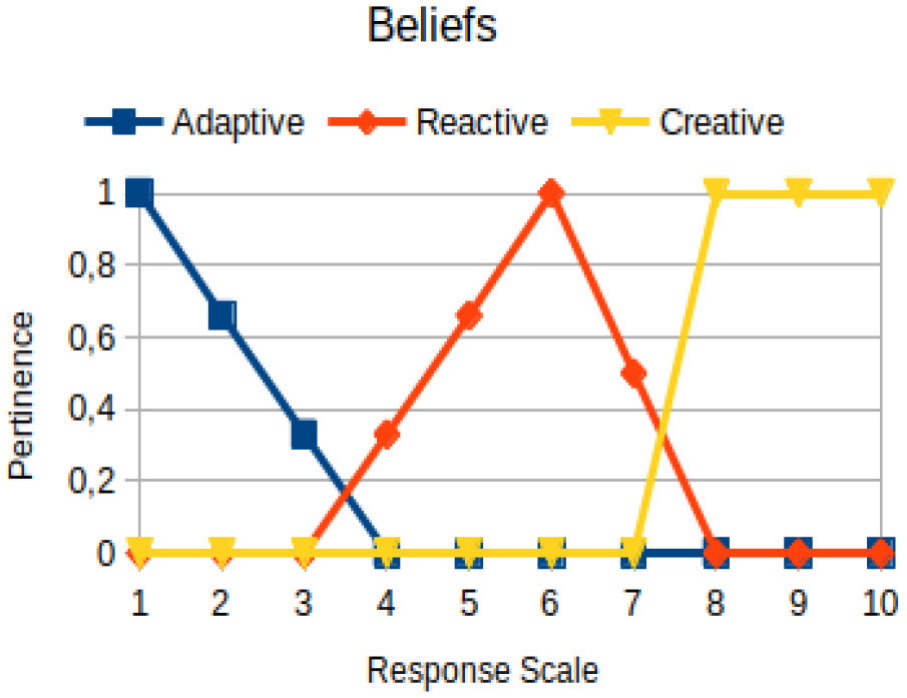
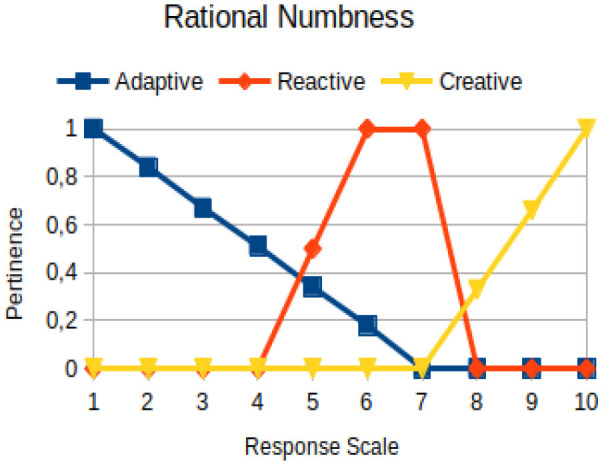	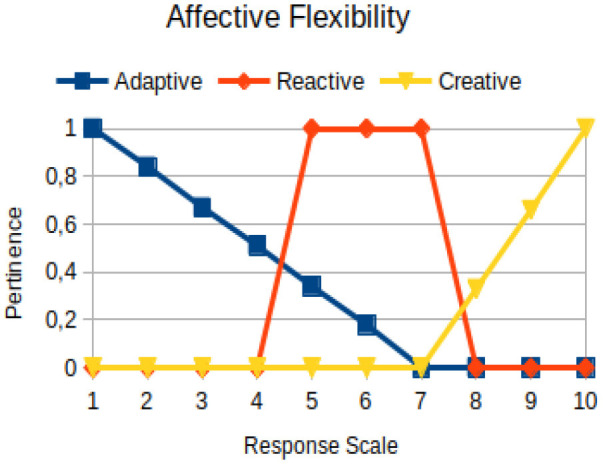
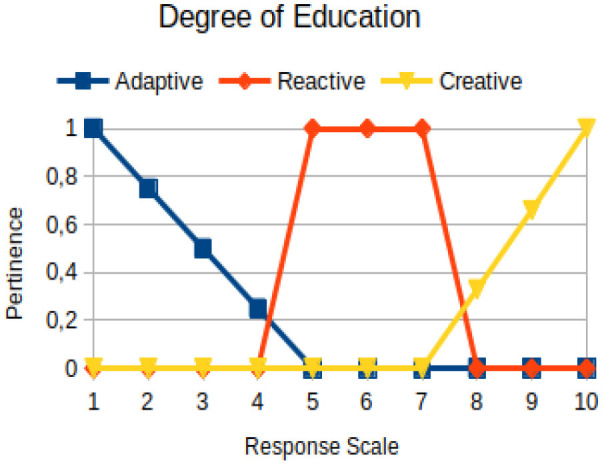	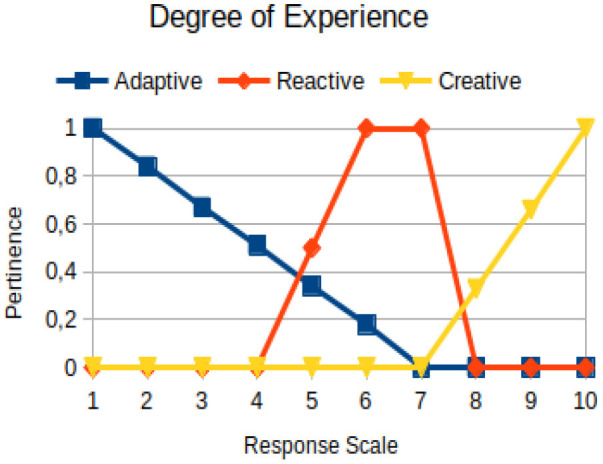
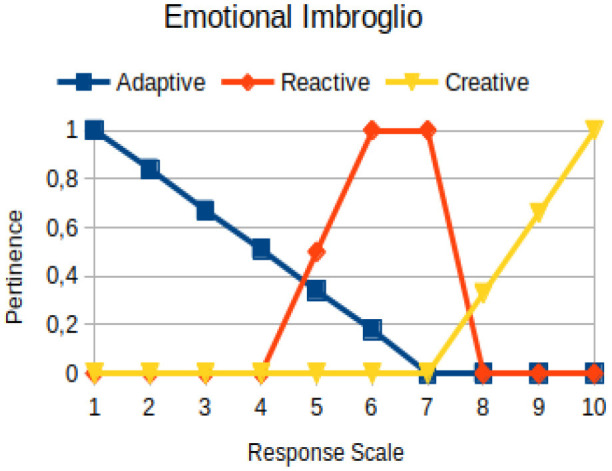	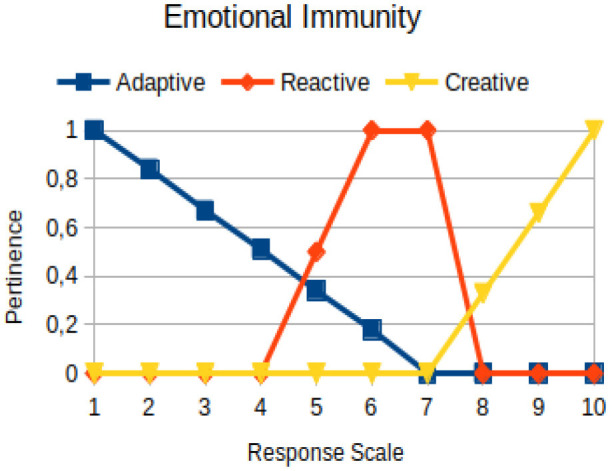
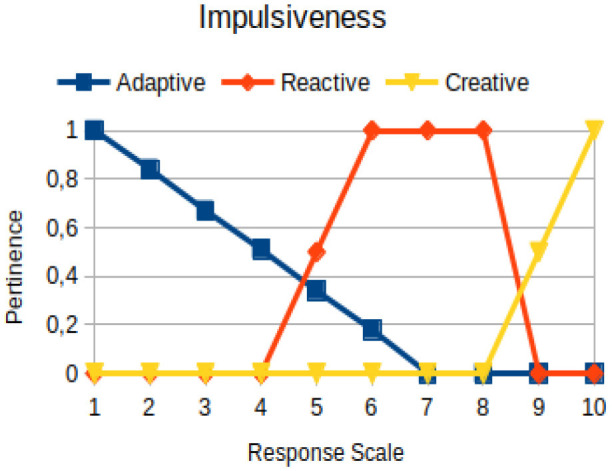	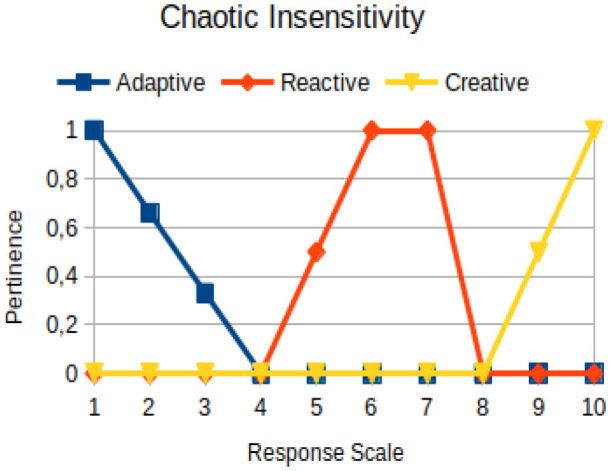
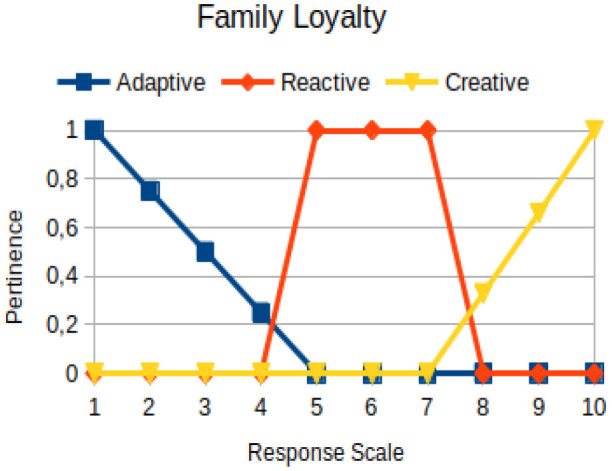	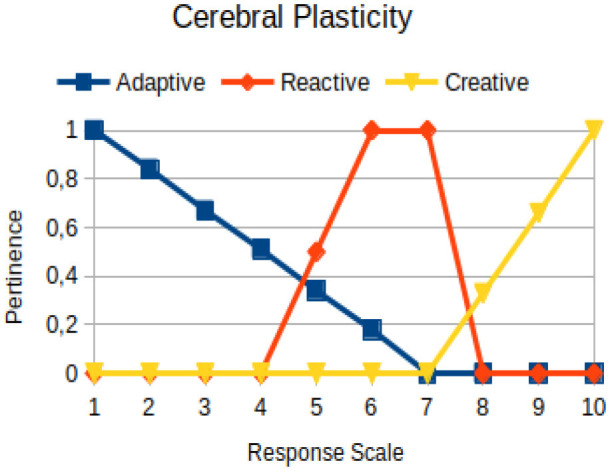
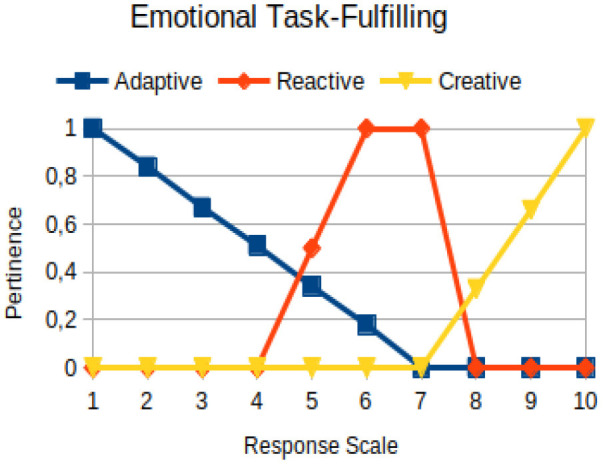	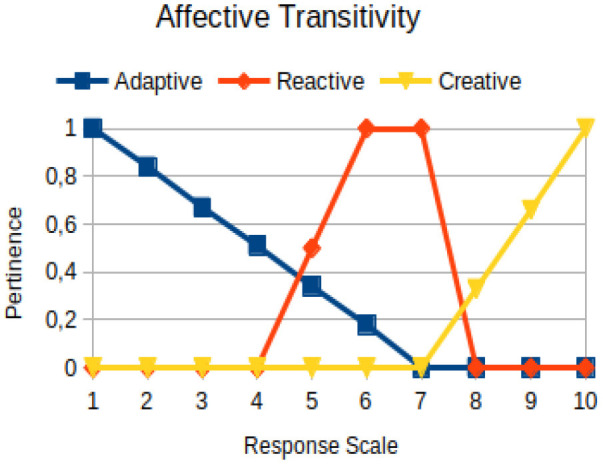


## Machine Learning

The technique for classifying mental functioning pattern profiles employs the k-nearest neighbors (KNN) algorithm (Altman, 1992). It is a classic AI approach with supervised machine learning, implemented using the scikit library (Pedregosa et al., 2011). Machine learning using KNN requires a sample of instances, each with its own set of characteristics. In addition, each of these instances is associated with an expected result (target), which is ultimately the subject to be predicted. The machine takes these instances with their characteristics and finds a mathematical function that makes it possible to group instances with the same target. When this mathematical function is found, known as the prediction model, the stage called training is finished and the machine is able to infer the targets of new instances. In this study, instances are represented by patients; the characteristics of the instances are the mental functioning patterns related to each of the categories (primary and circumstantial) of interest organized in a vector of patterns; and the target is the decision made by the patient. These items can be seen in the following figure.

**Figure 1:**
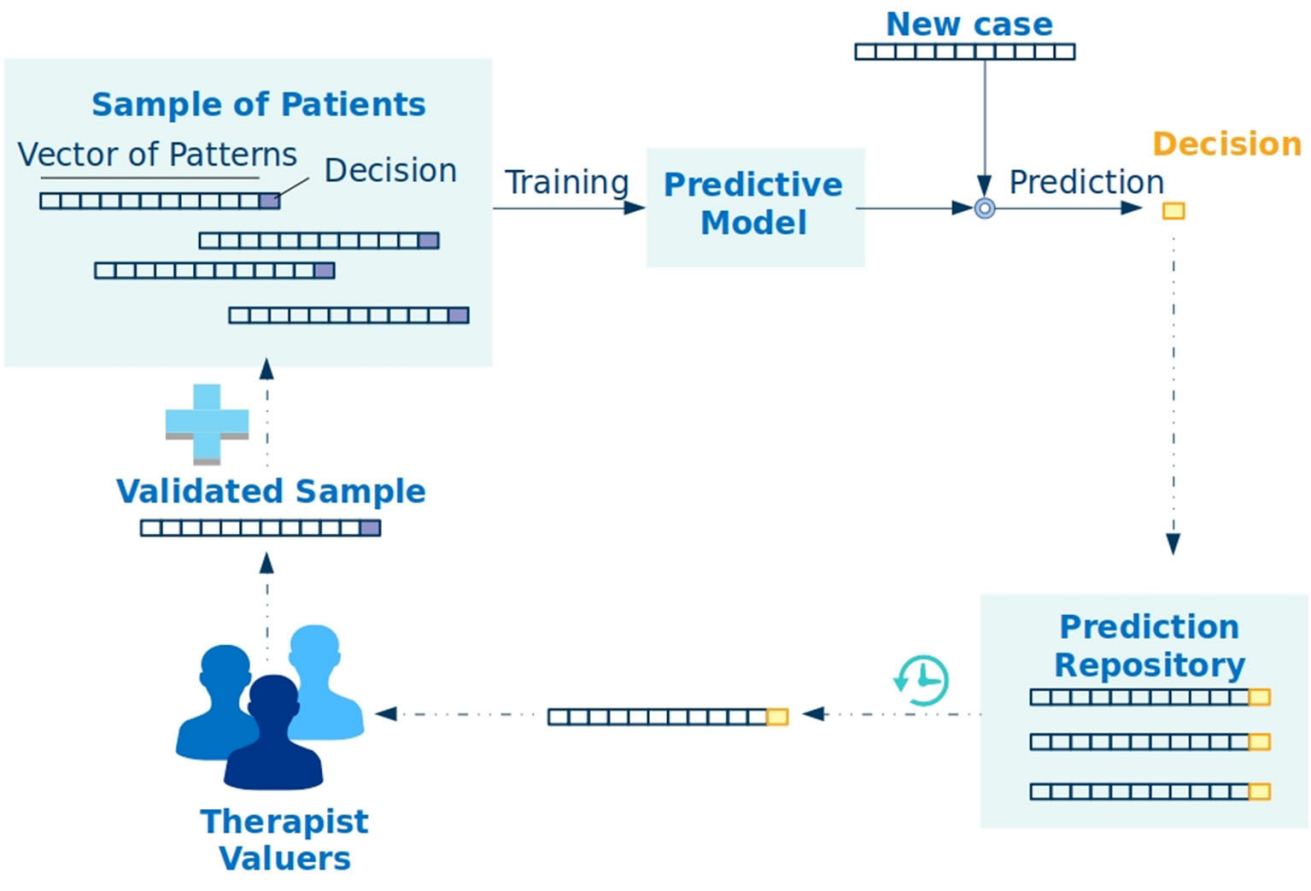
The iterative process of machine learning and inference of patient decisions.

Figure 1 shows the iterative process of discovering the likely decisions of a patient. The process begins with a sample of patients containing the vector of mental functioning pattern and the decision of each patient. Note that the decision-making scenario is dynamic and strongly coupled with the situation to be modeled. For this reason, the representative instance of the patient is formed by three distinct parts. The first contains the patterns of mental functioning associated with the primary categories (universal, innate, and pre-organized characteristics), the second part contains patterns associated with a selection of circumstantial categories (social characteristics), and the third contains the expected patient decision for the decision-making scenario. Note that the second and third parts of this representation are responsible for contextualizing the aspects of thematic interest that are to be measured, with the name circumstantial drivers being given to what shapes the thematic interest. In this way, the circumstantial drivers point to the theme that effectively expresses an influence on what is desired to be evaluated. Table 4 illustrates some circumstantial drivers and categories that offer information about patient behavior in the context being evaluated.

**Table 4:** Illustration of circumstantial drivers and their respective circumstantial categories of interest.

**Circumstantial drivers**	**Circumstantial categories of interest**
Beneficiary evaluation of health plan	Family environment, risk attachment, beliefs, degree of experience, family loyalty, cerebral plasticity
Debt collection	Financial literacy, family environment, assertiveness, self-control, beliefs, impulsiveness
Consumption of material goods	Financial literacy, family environment, assertiveness, self-control, self-esteem, self-image, rational numbness, affective flexibility, degree of education, degree of experience, emotional imbroglio, emotional immunity, impulsiveness, chaotic insensitivity, cerebral plasticity
Insurance proposal	Family environment, risk attachment, rational numbness, degree of education, emotional imbroglio
Selection process	Assertiveness, self-esteem, self-image, affective flexibility, degree of education, degree of experience, emotional immunity, impulsiveness, chaotic insensitivity, cerebral plasticity, affective transitivity
Personal/professional planning	Assertiveness, self-control, self-esteem, self-image, beliefs, affective flexibility, degree of education, degree of experience, emotional imbroglio, emotional immunity, impulsiveness, chaotic insensitivity, cerebral plasticity, emotional task-fulfilling, affective transitivity
Personal treatment	Assertiveness, self-esteem, affective flexibility, degree of experience, emotional immunity, family loyalty, cerebral plasticity, emotional task-fulfilling, affective transitivity

Once the patient sample of a circumstantial driver has been assembled, the machine is trained using the KNN algorithm, resulting in a prediction model. The system is then put into production to receive prediction requests for new patient cases. Strictly speaking, the objective of the system ends at the moment when the decision is predicted; however, in the procedure that was conceived, the prediction is the beginning of a system feedback step that makes it possible to incrementally improve the prediction capacity of the machine. Thus, pattern vector and decision prediction of a new patient are stored in a prediction repository. The instances stored in this repository are periodically evaluated by psychologists and other validators who ratify, or rectify, decisions suggested by the machine. This validated sample is now included in the sample of patients so that it can contribute to future training.

## Experiment Description

The investigation method of Pierce was chosen in this study to validate the procedure of finding the profile of a decision maker, based on emotional aspects, in order to determine a probable decision. In the abductive stage, an explanatory hypothesis is created for the decision-making process. In the deductive stage, an association is built between the characteristics of a patient and the likelihood that he/she would respond/behave in a certain way. In the inductive stage, the deduction test is performed with two explanatory hypotheses being put to the test: (1) The behavior of a person can be approximated by an indicator such as the mental functioning pattern; and (2) the mental functioning pattern can signal the most likely decisions of an individual.

The hypothesis that the behavior of a person can be approximated by an indicator such as mental functioning pattern was first validated by constructing the mental functioning pattern of several patients, which were then confronted with the perception of psychologists about the behavior and attitudes of these patients. In this sense, an online questionnaire was prepared containing the seven questions related to the primary categories of individuals (universal, innate, and pre-organized). The answers to these questions were modulated according to the scheme described in section The Profiling Method: Specific Mental Functioning Pattern of this study and used to produce the pattern vector of a person. The dominance of one pattern (adaptation, reaction, or creativity) over the others suggests a certain type of behavior, as described by Mello and Souza (2019) and discussed in section The Profiling Method: Specific Mental Functioning Pattern herein. These approximate suggestions for the behavior of an individual were presented to a psychologist, who was then able to use his/her professional patient assessment to agree, or not, with the suggestion.

The questionnaire was available for responses between March 30 and April 5, 2020. A total of 148 patients and former patients of a family therapy clinic-school responded to the questionnaire. The age of the individuals ranged from 17 to 73 years, and there were 91 women and 43 men who fit into 51 professional occupations. The questionnaire and the resulting dataset are available as Supplemental Material in the Frontiers portal.

Professionals with proven clinical experience were chosen as evaluating psychologists. Each psychologist was able to freely select, among the 148 individuals who answered the questionnaire, those they had previously worked with therapeutically, which allowed them to have a more accurate view of the behavior of an individual. The psychologists also excluded from their list those individuals who are part of their own personal social group, given the potential for interference by the emotional factor when evaluating a person who is part of the circle of relationships, thus avoiding contamination by their beliefs, values, and preconceptions about the person.

The values calculated for the mental pattern of a person were organized in a vector of three coordinates vc = (adaptive, reactive, and creative). In a similar way, a vp vector was created that represents the values given by the psychologist for the mental functioning pattern of the patient. The similarity between the calculated mental functioning pattern and that provided by the psychologist was assessed using cosine similarity as a metric (Baeza-Yates and Ribeiro-Neto, 2011). This technique is commonly used as a measure of similarity in tasks of data mining, information retrieval, and AI. When the cosine of the angle between vectors is 1, there is maximum similarity, whereas vectors with cosine 0 have no similarity. This study adopted the value of 0.8 as a threshold for accepting similarity between responses. Table 5 presents the vectors of the mental functioning patterns determined by calculation and psychologist evaluation, as well as the similarity between such vectors and the number of disagreements.

**Table 5:** Cosine similarity between the mental functioning pattern calculated by the proposed method and that from the evaluation by a psychologist.

	**Calculated (v**_****c****_ **)**			**Psychologist (v**_****p****_ **)**				
**Patient**	**Adaptive**	**Reactive**	**Creative**	**Adaptive**	**Reactive**	**Creative**	**Similarity**	**Number of disagreements**
1	0.073	0.605	0.322	0.469	0.344	0.188	0.718	1
2	0.132	0.279	0.588	0.467	0.333	0.200	0.675	1
3	0.352	0.267	0.381	0.571	0.286	0.143	0.869	0
4	0.322	0.194	0.483	0.444	0.444	0.111	0.724	1
5	0.017	0.914	0.069	0.375	0.500	0.125	0.808	0
6	0.016	0.744	0.240	0.250	0.500	0.250	0.910	0
7	0.085	0.740	0.175	0.429	0.429	0.143	0.794	1
8	0.000	0.950	0.050	0.571	0.286	0.143	0.447	3
9	0.000	0.978	0.022	0.400	0.400	0.200	0.674	1
10	0.019	0.444	0.537	0.500	0.375	0.125	0.547	4
11	0.151	0.672	0.177	0.471	0.412	0.118	0.815	0
12	0.353	0.197	0.451	0.500	0.375	0.125	0.794	1
13	0.513	0.162	0.325	0.457	0.371	0.171	0.909	0
14	0.245	0.527	0.228	0.400	0.400	0.200	0.946	0
15	0.081	0.163	0.756	0.500	0.375	0.125	0.396	5
16	0.000	0.693	0.307	0.278	0.500	0.222	0.892	0
17	0.079	0.659	0.261	0.500	0.313	0.188	0.667	2
18	0.232	0.000	0.768	0.467	0.400	0.133	0.418	6
19	0.000	0.000	1.000	0.467	0.400	0.133	0.212	5
20	0.101	0.378	0.521	0.500	0.438	0.063	0.571	4
21	0.000	1.000	0.000	0.400	0.400	0.200	0.667	2
22	0.075	0.721	0.203	0.471	0.412	0.118	0.744	1
23	0.000	0.501	0.499	0.571	0.357	0.071	0.448	2
24	0.532	0.345	0.123	0.357	0.500	0.143	0.933	2
25	0.105	0.350	0.545	0.471	0.353	0.176	0.668	2
26	0.020	0.509	0.470	0.438	0.500	0.063	0.633	4
27	0.144	0.711	0.144	0.444	0.389	0.167	0.803	0
28	0.156	0.022	0.822	0.438	0.500	0.063	0.234	6
29	0.118	0.314	0.568	0.429	0.500	0.071	0.567	2
30	0.072	0.271	0.657	0.438	0.500	0.063	0.436	4
31	0.052	0.819	0.130	0.444	0.444	0.111	0.757	1
32	0.714	0.000	0.286	0.471	0.353	0.176	0.818	0
33	0.191	0.222	0.587	0.462	0.462	0.077	0.547	4
34	0.020	0.589	0.391	0.375	0.500	0.125	0.778	1

Ideally, the similarity between the values of vc and vp should be within the acceptance range, which would immediately validate the tested hypothesis. However, Table 5 presents cases for which the similarity does not meet the acceptance criteria, which opens up the possibility of two different scenarios: (1) The psychologist did not correctly evaluate the patient; or (2) the patient did not answer the questionnaire correctly. In this sense, the evaluations produced by the psychologist were analyzed and no flaws were found in the values estimated by the psychologist. For this reason, the focus of attention shifted to the patients and their responses. An analysis was made for each answer given by the patient to the questions on the form, and several responses were found to differ by the behavior externalized by the patient; in other words, there was no consistency on the part of the patient in these cases.

Table 5 presents a count of questions for which there was a disagreement and indicates that a harmful impact on the similarity measure is associated with an increase in the number of disagreements. There are two reasons for the occurrence of these disagreements in the responses given to the questions on the forms: (1) There is an unconscious distortion between how the individuals perceive themself and how they are perceived by others, and (2) there is a conscious intention to answer the questions so as to signal a more mature mental behavior than actually possessed by the patient. When disagreements are resolved, mental functioning patterns become highly similar, which validate the hypothesis that the behavior of a person can be approximated by this pattern.

Then, the hypothesis of whether a mental functioning pattern can signal the most likely decisions of an individual was evaluated. This was carried out by using that pattern to indicate the likely decision and confront it with the decision that was actually made and executed by the individual. This stage of the experiment was carried out in a non-clinical environment, in partnership with a debt collection company related to cable TV subscriptions and internet access, respecting Brazilian General Data Protection Law (LGPD, English translation). This context selects the debt collection driver, described in Table 4, which contains six circumstantial interest categories to be evaluated. Added to this driver were the primary categories: self-esteem, self-discipline, anxiety, and learned resilience.

Despite the following experiment involves the negotiation of debit collection, the method described in this study is applicable to other decision-making domains, such as the ones from Table 4. The reader must be aware that the coming scenario is used to support and validate our hypothesis, providing a real environment, not an artificially created one.

The partner company in this experiment had a computerized and automated debt collection system, where debtors can decide whether or not to make an agreement, according to discount and deadline criteria suggested by the system. Prior to the start of the negotiation, a questionnaire with 10 questions related to the mental functioning pattern was introduced into the debt collection system, which makes it possible to indicate the expected behavior of a debtor when making an agreement. Later, and offline, this expected value of the agreement was compared with the agreement that was actually chosen by the debtor and the degree of fulfillment of this obligation. The variables of choices of the debtor used in the experiment were as follows: “agreement made or not,” “discount amount for debt settlement,” “number of installments for payment of the debt,” and “number of installments actually paid.”

Debtors were free to choose whether or not to participate in the experiment by providing consent on a website. The questionnaire was available for responses between April 14, 2019, and May 9, 2019. A total of 1,204 debtors, 592 women, and 612 men aged 18–91 years answered the questionnaire, whose debts were on an average 178 days old and had an average liability of R$ 713.17 (180.22 USD). The questionnaire and the resulting dataset are available as Supplemental Material in the Frontiers portal.

The data were organized in a Vector of Patterns with 10 positions, each associated with one of the evaluation questions asked to the debtor and whose content contained records of the responses obtained (see Table 6). Then, a cell representing the agreement decision chosen by the debtor was added to the vector (see Figure 1). In this way, a dataset of 1,204 elements was created, which was divided into sets for training, testing, and validation with 70, 18, and 12%, respectively, of the samples chosen at random. The predictive model resulting from the training was obtained using the KNN clustering technique (Altman, 1992), which is generally a good technique for prediction problems of this nature.

**Table 6:** Questions and linguistic terms used for the evaluated metrics.

**Question**	**Linguistic terms**
Anxiety Are you a person that is in a constant state of alert?	To the left: No Center: Sometimes To the right: Yes
Self-discipline Do you consider yourself a disciplined and organized person to practice actions that lead to your goal?	To the left: No Center: It depends To the right: Yes
Self-esteem Are you a person who recognizes your qualities and acts to express your feelings and behaviors?	To the left: I do not recognize or act Center: Sometimes I recognize and act To the right: I recognize and act
Beliefs When making decisions do you seek to base yourself on the values and habits learned in your family of origin?	To the left: Yes Center: Sometimes To the right: No
Cultural empowerment In your opinion, does culture influence decision-making?	To the left: No Center: It depends To the right: Yes
Cognitive flexibility How much capacity do you think you have to change your beliefs, paradigms, and values in the face of adversity?	To the left: Low Center: Medium To the right: High
Cognitive preservation Do you imagine that you have some difficulty to perceive and act in the face of obstacles of life?	To the left: Yes Center: Depends on the situation To the right: No
Learned resilience How much can you learn from your experiences?	To the left: Little Center: What is necessary To the right: Very
Financial literacy Do you consider yourself a person who always balances your income/expenses against your needs?	To the left: No Center: Sometimes To the right: Yes
Family environment How is money used, that is, the value it is given in your family of origin?	To the left: Limited Center: It depends To the right: Free
Risk attachment Do you consider yourself a person who generally assesses the risk you face when facing challenges?	To the left: No Center: It depends To the right: Yes
Assertiveness Are you a person who recognizes your qualities and acts to express your feelings and behaviors?	To the left: I do not recognize or act Center: Sometimes I recognize and act To the right: I recognize and act
Self-control What do you use when making a decision?	To the left: More emotion Center: Emotion and reason To the right: More reason
Self-image Is the image you convey to people consistent and authentic with your real image?	To the left: No Center: It depends To the right: Yes
Rational numbness In general, when you have to make a decision, do you try to make use of reason and emotion, or in most people do not worry about it?	To the left: Emotion Center: It depends To the right: Reason and emotion
Affective flexibility Can you see learning when you experience frustration, failure, or failure in your personal or professional life?	To the left: No Center: It depends To the right: Yes
Degree of education Do you think that knowledge has been a source of growth in your personal and professional life?	To the left: No Center: It depends To the right: Yes
Degree of experience Do you imagine that a person with countless experiences has the capacity to make better choices for you?	To the left: No Center: It depends To the right: Yes
Emotional imbroglio In your opinion, do material, financial, and emotional difficulties experienced by a person interfere with decision- making?	To the left: No Center: It depends To the right: Yes
Emotional immunity Are you a person who seeks to learn from the experience of the other, when arguments of the other are well-founded, even if you have to give up your convictions?	To the left: No Center: It depends To the right: Yes
Impulsiveness How do you assess your reaction to everyday issues?	To the left: Elaborated Center: It depends To the right: Immediate
Chaotic insensitivity Do you usually notice something that has bothered you in a new environment and does something to change this situation?	To the left: No Center: It depends To the right: Yes
Family loyalty Can a loyal person consciously make decisions with “the eyes of family members?”	To the left: No Center: It depends To the right: Yes
Cerebral plasticity In general, are you a person who accepts new information to make the necessary changes in order to change your quality of life?	To the left: No Center: It depends To the right: Yes
Emotional task-fulfilling In general, are you a person who recognizes and expresses your emotions in your activities as a whole?	To the left: No Center: It depends To the right: Yes
Affective transitivity Are you a person who moves between different groups using flexibility to get out and get into various interpretive roles?	To the left: No Center: It depends To the right: Yes

Table 7 consolidates the results obtained from predictions with the validation set, that is, the predictive model was produced with the training and testing set, but the definitive evaluation of the model was carried out with the validation set. The decision variables represent the aspects to be predicted, the enumeration of responses represents the possible choices that the debtor can make, the number of classes represents the count of different choices available to the debtor, the success rate by random choice represents the expected value of correct predictions if they were made by a random criterion, the success rate of prediction model represents the percentage of correct predictions made by the model based on the mental functioning pattern, and the gain is the relative value between the correct prediction model and the random choice. Computer scientists may be interested in the prediction model accuracy, precision, and recall presented at Table 8. It shows the mean value between labels of each model.

**Table 7:** Results for predicting decisions of a debtor according to profiling through mental functioning patterns.

**Decision variable**	**Enumeration of responses**	**Number of classes**	**High rate by random choice %**	**High rate by prediction model %**	**Gain**
Agreement was reached	Yes, now	2	50.00	60.14	1.2028
Discount for debt settlement	0, 15, 20, 25, 30, 35, 40, 45, 50, 55, 60, 65, 70, 75, 80	15	6.67	46.74	7.0075
Number of installments to pay the debt	1, 2, 3, 4, 5, 6	6	16.67	56.06	3.3629
Number of installments actually paid	0, 1, 2, 3, 4	5	20.00	49.86	2.4930

**Table 8:** Prediction model evaluators.

**Prediction model**	**Accuracy %**	**Precision %**	**Recall %**
Agreement was reached	60.14	78.06	69.78
Discount for debt settlement	46.74	61.18	58.71
Number of installments to pay the debt	56.06	69.97	68.08
Number of installments actually paid	49.86	68.11	60.75

The gain in signaling the most likely decision of the debtor related to the discount amount is in the order of 7.0075 times greater than that of a random prediction, which is a very significant amount. The gains for the predictions of the variables of “number of installments chosen for payment” and “number of installments actually paid” are 3.3629 and 2.4930 times greater, respectively, than the random prediction, which are also significant values. The gain in “discovering the effectiveness of the agreement” is lower, at 1.2028 times, but despite being overshadowed by the other gains, it is still a considerable amount. These results unequivocally suggest validation of the hypothesis that mental functioning pattern can signal the most likely decisions of an individual.

In addition, it is also worth noting some relevant points. Under the perspective of a machine learning study, the 1,204-sample dataset is still considered small. Small datasets may produce overfitting predictive models that would consequently perform poorly in predicting new data. The gains obtained in validation were high, and so it is reasonable to expect that there is room to increase them further, which is interesting in the case of the variable of “discovering the effectiveness of the agreement.”

## Conclusions

This study presented a procedure to determine the profile of a decision maker based on psychological and emotional aspects, made operational by AI techniques. The two proposed hypotheses were confirmed: (1) Behavior of a person can be approximated by an indicator such as mental functioning pattern; and (2) mental functioning pattern can signal the most likely decisions of an individual. In this way, understanding the specific mental functioning pattern of a person increases the ability to predict his/her actions, facilitates the task of inducing his/her behavior, and helps in patient awareness work. Therefore, knowing the specific mental functioning pattern of an individual is a valuable asset for the clinical area and for all other areas of psychology, and the modeling proposed here is an effective tool for this process.

It will be necessary, in future studies, to increase the training dataset for predictive models to improve the quality of machine learning classifiers. Here, seven circumstantial drivers were proposed, but only one was worked with, and so new experiments can be conducted to study the other drivers. Another point that seems interesting to study is the sensitivity of the procedure presented here in relation to the sociogeographic space of other nationalities, as there is an expectation, although without experimentation, that mental functioning patterns differ in relation to this characteristic.

This study can direct the construction of software for understanding and mapping the specific mental functioning pattern of individuals or groups. Whatever the purpose of such application, it will be necessary to develop a code of ethics for the availability and use of such computational tools.

## Data Availability Statement

The raw data supporting the conclusions of this article will be made available by the authors, without undue reservation.

## Ethics Statement

This research was submitted to Brazilian ethics committee plataform (Plataforma Brasil) according Brazilian regulation, reviewed and approved by CEP/IPUB (Comitê de Ética em Pesquisa/Instituto de Psiquiatria da UFRJ—Research Ethics Committee/UFRJ's Psychiatry Institute), and with registration number CAAE 90390518.2.0000.5263. The patients/participants provided their written informed consent to participate in this study.

## Author Contributions

All authors listed have made a substantial, direct and intellectual contribution to the work, and approved it for publication.

## Conflict of Interest

The authors declare that the research was conducted in the absence of any commercial or financial relationships that could be construed as a potential conflict of interest.

## Publisher's Note

All claims expressed in this article are solely those of the authors and do not necessarily represent those of their affiliated organizations, or those of the publisher, the editors and the reviewers. Any product that may be evaluated in this article, or claim that may be made by its manufacturer, is not guaranteed or endorsed by the publisher.
