# Supplementary material for: Decision Maker Profiling Using Their Mental Behavior Pattern
Source: Front Psychol. 2021 Aug 18;12:667255. doi: 10.3389/fpsyg.2021.667255 (PMC8416518; doi:10.3389/fpsyg.2021.667255)
Supplement: Supplementary file 2 [file Data_Sheet_2.pdf]

Hello dear friend,

We are working on a survey to investigate some behavior patterns and relate them to the way people act when making decisions.

Could you help us by answering these questions? You will spend a maximum of 2 minutes.

Thanks,

Sebastião/Flávio

Name (small text)

Age (small text)

When having to make a decision, do you believe you will encounter any difficulties?
Yes 1 2 3 4 5 6 7 8 9 10 No

How would you rate this difficulty?
Low 1 2 3 4 5 6 7 8 9 10 High

Are you a person in constant state of alert?
No 1 2 3 4 5 6 7 8 9 10 Yes

You are a person who recognizes your qualities and acts in a way that expresses the your feelings and behaviors?
Neither Recognize, nor Act 1 2 3 4 5 6 7 8 9 10 Recognize and Act

Do you consider yourself a disciplined and organized person to take actions that lead to your goal?
No 1 2 3 4 5 6 7 8 9 10 Yes

Do you imagine that you have any difficulty in perceiving and acting when facing obstacles?
Yes 1 2 3 4 5 6 7 8 9 10 No

How much can you learn from your lived experiences?
Little 1 2 3 4 5 6 7 8 9 10 Much

In your opinion, does culture influence decision making?
Yes 1 2 3 4 5 6 7 8 9 10 No
