# Supplementary material for: Decision Maker Profiling Using Their Mental Behavior Pattern
Source: Front Psychol. 2021 Aug 18;12:667255. doi: 10.3389/fpsyg.2021.667255 (PMC8416518; doi:10.3389/fpsyg.2021.667255)
Supplement: Supplementary file 4 [file Data_Sheet_4.pdf]

OVERDUE CLIENTS BEHAVIOR PATTERNS

1 - What is your date of birth?

2 - How old is the debt? (information already in MFM DB)

3 – Do you consider yourself a person who makes decisions easily?
(C) Yes
(A) No
(R) So-so

4 – Do you consider yourself a disciplined person focused on what you want to achieve?
(C) Yes
(A) No
(R) So-so

5 – How do you react when you get compliments (generally thinking)?
(A) I like it and I feel fulfilled
(R) I like it more or less
(C) It depends on where this compliment comes from

6 – Do you consider yourself an optimistic or pessimistic person?
(A) Optimistic
(R) Pessimistic
(C) Neither optimistic nor pessimistic

7 – In your interpersonal relationships (dating, partnerships, marriages) and professionals, do you choose more or are you chosen?
(C) In general, I choose
(A) I am chosen
(R) It depends on the situation

8 – What can be said about your earnings and your expenses?
(R) Controlled but sometimes I get lost
(C) Planned and organized
(A) I buy what I need to meet my needs

9 – How do you buy the things you like?
(C) I make a spreadsheet of my expenses
(R) I buy and adjust the way to pay
(A) I buy because I like it and I know I can pay

10 – When you are going to make a purchase, how do you know you will be able to pay?
(C) I know I have a cash reserve
(R) I know what I earn and spend, and I know I can adjust it if needed
(A) I know because my effort always fulfill what I need

11 – Did your parents talk to you about how to take care of your money?
(A) In general not
(R) More or Less
(C) Yes, they talked

12 – Were your parents people who valued the acquisition of material and financial goods?
(C) Yes, they valued
(R) For some things yes, others not
(A) No, this was not so important for our life

13 – What was the reason for your indebtedness? <months to recover>
<3-6> ( ) Unemployment
<12> ( ) Death of person who provided financial support
<18> ( ) Illness in the family
<6-12> ( ) Family dissolution
<6-12> ( ) Family conflict
<6-12> ( ) Addiction
<6> ( ) I assumed the debt of third parties
<0> ( ) Others: Which ones?
